# Direct and Sensitive Detection of Dopamine Using Carbon Quantum Dots Based Refractive Index Surface Plasmon Resonance Sensor

**DOI:** 10.3390/nano12111799

**Published:** 2022-05-25

**Authors:** Faten Bashar Kamal Eddin, Yap Wing Fen, Nurul Illya Muhamad Fauzi, Wan Mohd Ebtisyam Mustaqim Mohd Daniyal, Nur Alia Sheh Omar, Muhammad Fahmi Anuar, Hazwani Suhaila Hashim, Amir Reza Sadrolhosseini, Huda Abdullah

**Affiliations:** 1 Department of Physics, Faculty of Science, Universiti Putra Malaysia (UPM), Serdang 43400, Selangor, Malaysia; gs51801@student.upm.edu.my (F.B.K.E.); nuralia_so@upm.edu.my (N.A.S.O.); gs56100@student.upm.edu.my (H.S.H.); 2 Functional Nanotechnology Devices Laboratory, Institute of Nanoscience and Nanotechnology, Universiti Putra Malaysia (UPM), Serdang 43400, Selangor, Malaysia; gs55644@student.upm.edu.my (N.I.M.F.); gs50207@student.upm.edu.my (W.M.E.M.M.D.); gs59097@student.upm.edu.my (M.F.A.); 3 Magneto-Plasmonic Laboratory, Laser and Plasma Research Institute, Shahid Beheshti University, Tehran 1983969411, Iran; a_sadrolhosseini@sbu.ac.ir; 4 Department of Electrical, Electronic and Systems Engineering, Faculty of Engineering and Built Environment, Universiti Kebangsaan Malaysia, Bangi 43600, Selangor, Malaysia; huda.abdullah@ukm.edu.my

**Keywords:** dopamine, neurotransmitters, surface plasmon resonance, optical sensor, carbon quantum dots, refractive index sensor, sensitivity enhancement

## Abstract

Abnormality of dopamine (DA), a vital neurotransmitter in the brain’s neuronal pathways, causes several neurological diseases. Rapid and sensitive sensors for DA detection are required for early diagnosis of such disorders. Herein, a carbon quantum dot (CQD)-based refractive index surface plasmon resonance (SPR) sensor was designed. The sensor performance was evaluated for various concentrations of DA. Increasing DA levels yielded blue-shifted SPR dips. The experimental findings revealed an excellent sensitivity response of 0.138°/pM in a linear range from 0.001 to 100 pM and a high binding affinity of 6.234 TM^−1^. The effects of varied concentrations of DA on the optical characteristics of CQD thin film were further proved theoretically. Increased DA levels decreased the thickness and real part of the refractive index of CQD film, according to fitting results. Furthermore, the observed reduction in surface roughness using AFM demonstrated that DA was bound to the sensor layer. This, in turn, explained the blue shift in SPR reflectance curves. This optical sensor offers great potential as a trustworthy solution for direct measurement due to its simple construction, high sensitivity, and other sensing features.

## 1. Introduction

Dopamine (DA) is a catecholamine neurotransmitter that is produced by neurons in the brain and plays a crucial role in the transmission of neurological signals. DA has a substantial impact on the functions of the human metabolism, central nervous system, and renal and hormonal systems. Some neurological diseases such as Parkinson’s disease and schizophrenia are caused by DA deficiency [1,2,3]. The physiological levels of DA in different human biofluids vary. According to the Human Metabolome Database, the concentration of DA in blood is less than 130 pM, whereas it is 5 nM in human cerebrospinal fluid and urine [4]. Therefore, the sensitive and rapid detection of very low concentrations of DA is critically needed and receiving a lot of attention in clinical diagnostics. Up to now, many methods have been conducted to detect DA levels, including high-performance liquid chromatography (HPLC) [5,6], fluorescence [7,8,9,10], chemiluminescence [11,12], mass spectrometry [13], capillary electrophoresis [14], and electrochemical [15,16] and surface plasmon resonance (SPR) [17,18,19,20,21,22,23,24,25,26,27,28,29,30]. Among them, SPR has recently emerged as a promising technique for the rapid detection of DA molecules, with the potential to reduce the challenges associated with many interfering chemicals. To this day, the use of SPR sensors to detect DA is quite limited, although the published results are encouraging. SPR biosensors have proven their efficacy due to their capacity to monitor diverse biomolecular interactions label free and in real time, as well as their fast response and accuracy, and excellent performance [31,32,33,34,35,36,37,38]. SPR sensors are particularly sensitive to boundary conditions and may detect even small variations in the surrounding refractive index caused by interactions between the analyte in solution and the SPR sensor thin film [39,40,41,42,43,44]. Therefore, the theoretical analysis enables understanding and prediction of the responses of plasmonic-based systems as a function of their microscopic properties, which may be crucial and provide a guideline for the precise control of the design of SPR-based sensors [45,46,47]. However, to monitor normal or extremely low levels of DA solution using an SPR sensor, the sensor’s sensitivity must be increased. To overcome this drawback, nanomaterials can be employed to modify the sensor chip. Carbon-based nanomaterials have a significant surface plasmon resonance, and surface plasmon resonance technology can enable it to break past the conventional optical diffraction limit, as well as demonstrate characteristics of local electromagnetic field augmentation, attaining perfect absorption [48]. Carbon quantum dots (CQDs), which are nanoparticles with extremely small sizes, typically less than 10 nm, have recently become widely used in a variety of applications, including biosensors, bio-imaging, drug delivery, cancer therapy, and bacterial infection control, due to their advantages, such as ease of preparation, low toxicity, good biocompatibility, and stability [49,50,51]. The use of CQDs in the preparation of SPR sensor chips for DA detection has not yet been reported. Based on these properties of CQDs, they were employed in this work as an active layer to improve the SPR sensor sensitivity to DA. The direct detection of DA with high sensitivity by the proposed sensor was demonstrated and the sensor performance was evaluated. In addition, the structural analysis of the sensor film in the absence and presence of DA was studied. Following that, the experimental SPR curves were mathematically processed in order to investigate the variation of the refractive index of the sensing medium as well as to analyze the optical properties of DA and CQD thin film, measure the thickness of CQD film, and determine the refractive index sensitivity of the proposed system. To the best of our knowledge, this is the first study on the detection of DA utilizing a CQD thin film-based SPR sensor.

## 2. Materials and Methods

### 2.1. Chemical Preparation

Dopamine hydrochloride and CQDs (0.2 mg/mL) with quantum efficiency (>5%) were supplied by Sigma-Aldrich. To make a 1 M concentration of dopamine aqueous solution, 4.741 g of dopamine hydrochloride were dissolved in 25 mL of deionized water (DW). Then, following the dilution formula (*M*_1_*V*_1_ = *M*_2_*V*_2_), DW was used to dilute the DA solution to produce extremely low values of 1 fM.

### 2.2. Sensor Chip Preparation 

Using the SC7640 Sputter Coater, gold thin films for SPR measurements were deposited on clean glass substrate surfaces with dimensions of 24 mm × 24 mm 0.1 mm. To produce the active layer, the surface of the gold thin layer on the glass substrate was evenly coated with 0.5 mL of 0.2 mg/mL CQD solution. Then, a thin layer of CQDs was obtained using the spin-coating process at 2000 rpm for 30 s.

### 2.3. Configuration of SPR System

**Figure 1:**
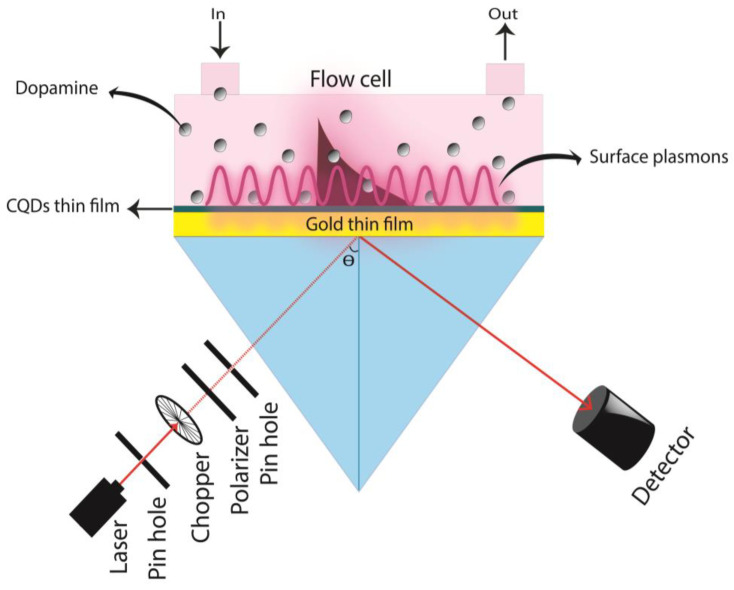
SPR configuration.

The ability of CQD thin film to detect DA was investigated using a custom-built SPR spectroscopy based on angle interrogation. The Kretschmann setup for surface plasmon wave resonance excitation was used with this handmade SPR sensor. As illustrated in Figure 1, it included a He-Ne laser with a wavelength of 632.8 nm, a light chopper, a linear polarizer, a tiny pinhole, a prism with refractive index of 1.77861, an optical rotating stage, a photodetector, and a lock-in amplifier. Using a matching gel, SPR chips were connected to the prism. SPR tests were performed, as well as angular spectral analysis, on gold thin films and CQD thin films that were exposed to DW and DA at varied concentrations. DW was introduced into the flow cell and made contact with the gold film and then the sensing layer one by one to get the reference signal. Thereafter, various concentrations of DA solution were gradually introduced into the flow cell in order to carry out the experiments, which included measuring the intensity of reflected light as a function of angle of incidence.

### 2.4. Structural Characterization Techniques

The FTIR spectra of CQD thin film before and after exposure to DA solution was recorded in the range of 400–4000 cm^−1^ using a Bruker ALPHA II FTIR Spectrometer in ATR mode. The Bruker AFM multimode 8 in Scan Asyst mode was used in the range of 2 × 2 μm for topographic imaging of all thin films and to analyze the roughness changes of CQD film after DA adsorption on its surfaces.

## 3. Results and Discussion

### 3.1. Structural Analysis

**Figure 2:**
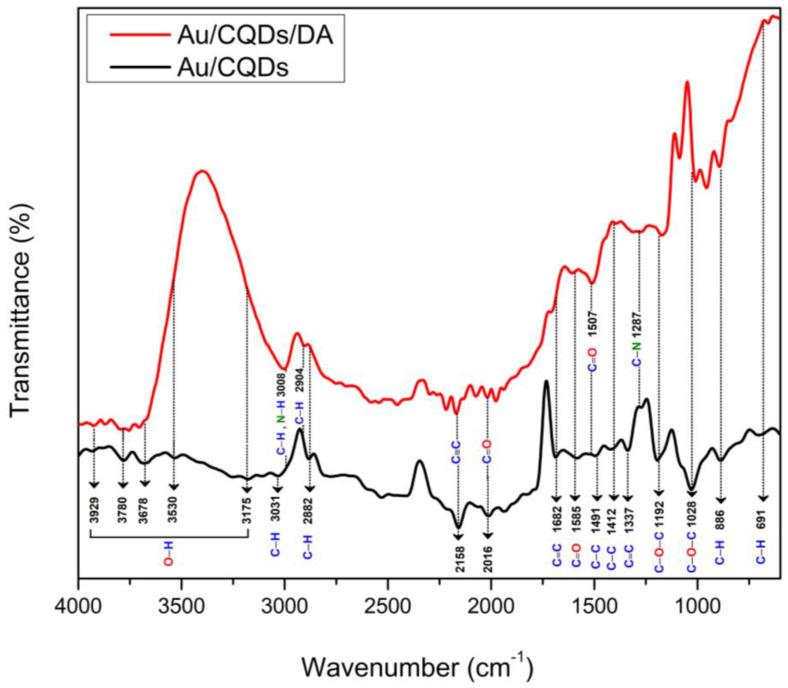
FTIR spectrum of CQD thin film before and after interaction with DA.

To analytically characterize CQD thin film before and after the adsorption of DA on its surface, FTIR spectra were recorded to elucidate the functional groups on the film surface. Figure 2 depicts the FTIR spectrum of CQD thin film (black spectrum), which was consistent with previous works and contained a variety of functional groups. The existence of abundant hydroxyl groups at the CQD surface was verified by the presence of the characteristic bands of O–H at the 3929, 3780, 3678, 3530 and 3175 cm^−1^ peaks [52,53,54,55,56]. The peaks appearing at 3031 and 2882 cm^−1^ were attributed to C–H stretching vibration [52,55,56,57,58,59,60]. The peak located around 2158 cm^−1^ corresponds to the stretching frequency of alkyne C≡C groups [61,62], and the peak at 2016 cm^−1^ originated due to C=O stretching of the carboxyl group [63]. The available peaks at 1682 and 1585 cm^−1^ were ascribed to C=C and C=O bond stretching [58,64]. In addition, the peaks appearing at 1491 and 1412 cm^−1^ were due to C–C stretching [65]. The peaks appearing at 1337 cm^−1^ originated due to C=C vibration [66]. The peaks at 1192 and 1028 cm^−1^ were attributed to C–O–C stretching vibrations [56,58,60,67], and the peaks located at 886 and 691 cm^−1^ corresponded to C–H bending vibrations [55]. The FTIR spectrum obtained for CQD film after exposure to DA (red spectrum) showed a reduction in the intensity of the O–H stretching band at 3780 cm^−1^ due to the overlap with the N–H stretching vibrations. It is also clear that the peak at 3031 cm^−1^ originating from C–H stretching vibrations was shifted to 3008 cm^−1^ and increased in intensity due to the –NH group. The peak located at 2882 cm^−1^ was shifted slightly to 2904 cm^−1^ due to alkyl C−H stretching [68]. The intensity of the observed peak around 1507 cm^−1^ was increased due to aromatic C=C stretching, and the peak located around 1287 cm^−1^ originated due to amine C–N stretching [68,69]. These findings confirmed the reaction between DA and CQD film and revealed that once DA was introduced, the functional groups of CQDs altered conformationally. This verifies that DA was bonded to the surface of the sensor film and was detected.

**Figure 3:**
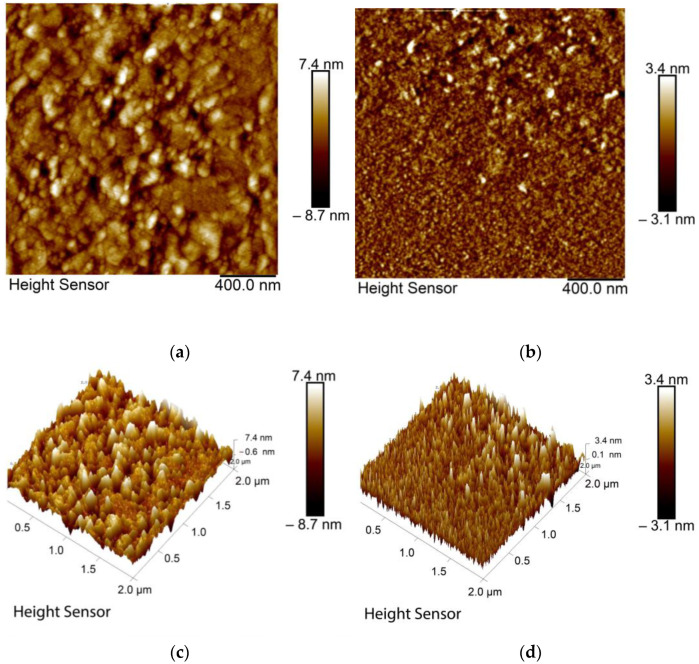
AFM images of CQD thin film. (**a**) 2D image before interaction with DA and (**b**) 2D image after interaction with DA. (**c**) 3D image before interaction with DA and (**d**) 3D image after interaction with DA.

AFM was used to continue structural characterization of the sensor film. The surface morphology of a CQD thin film before contact with DA is depicted in Figure 3a, where the 2D image shows the random distribution of CQDs on an Au substrate. The 3D AFM image (Figure 3c) of Au/CQD film in the absence of DA shows that the maximum height of CQD peaks was 7.4 nm, which is consistent with previous studies [70]. However, it is obvious from Figure 3b that DA adsorption on the sensor chip influenced the surface morphology. Furthermore, the presence of DA on the film surface made the detected peaks fewer and sharper, with a maximum height of 3.4 nm (Figure 3d). After DA injection, the average roughness of the sensor surface Ra was reduced from 1.60 nm to 0.642 nm, and the standard deviation of the Z values Rq, also known as the RMS roughness (root mean square), was reduced from 2.99 nm to 1.04 nm. These findings revealed that DA binding to CQD thin film strongly affected its morphology.

### 3.2. Detection of DA Using CQD-Based SPR Sensor

**Figure 4:**
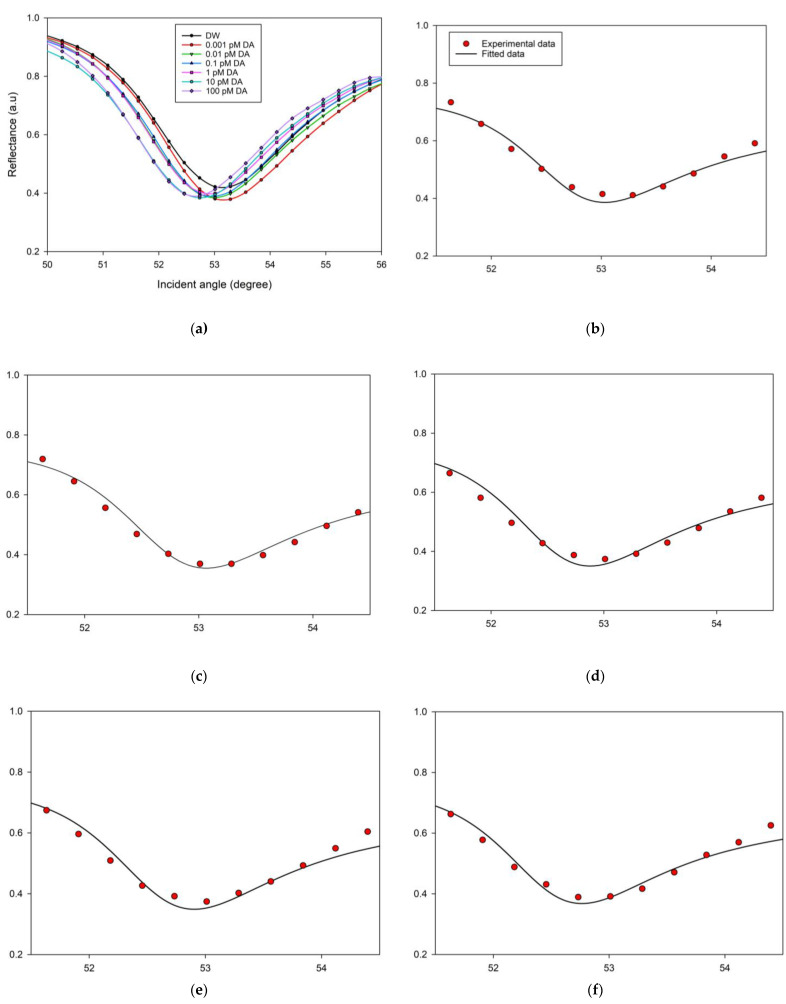

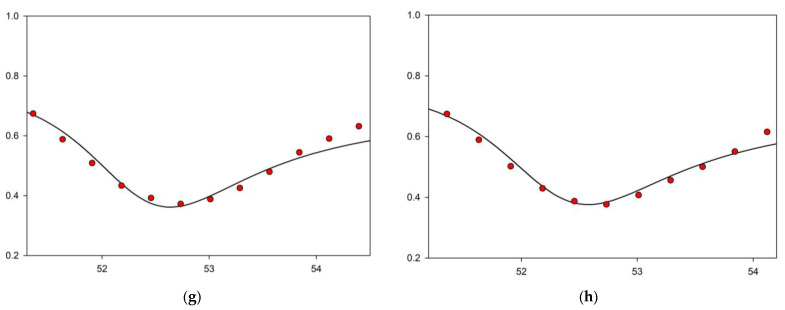
(**a**) Experimental SPR reflectivity curves related to CQD thin film exposed to different levels of DA solution; the experimental and fitted curves of the sensor film exposed to (**b**) 0 fM, (**c**) 1 fM, (**d**) 10 fM, (**e**) 100 fM, (**f**) 1 pM, (**g**) 10 pM, and (**h**) 100 pM of DA.

The measurements were done using DW and DA solution at various low concentrations ranging from 0.001 pM to 100 pM. When CQDS film made contact with DW, the resonance occurred at an angle of 53.28702°. By continuing the measurements, the resonance angle determined using SPR reflectivity curves for 0.001 pM of DA was 53.28647°, which is somewhat less than the angle obtained using DW, showing that the SPR dip underwent a blue shift compared to the reference signal. After increasing the DA level to 0.01 pM, the SPR dip was moved to 53.01033°, with a blue shift amounting to 0.27669° from the baseline. The introduction of a 0.1 pM DA solution made the resonance take place at angle of 53.01144°. Afterwards, DA concentration was raised to 1 pM and the resonance angle was conspicuously shifted to 52.73346°. The SPR dip stayed blue shifted while DA levels were increased to 10 pM, but the resonance angle altered by a very little step from the prior concentration’s SPR angle, as shown in Table 1. The same was true when detecting a DA concentration of 100 pM. Figure 4a depicts the recorded SPR spectra, which exhibited a blue shift as the concentration of DA increased. It may be observed that the SPR response curves did not appear to be shifted when high concentrations of DA were introduced. Since the sensing layer had a limited surface and hence a finite number of binding sites, this saturated the sensor response where the number of binding sites accessible per DA molecule reduced as its concentration in the sample solution increased.

The blue shift can be explained by the changes in the refractive indices and thickness of the sensing medium [71,72,73,74,75,76]. To determine the refractive index and the thickness of the Au/CQD thin film, the SPR experimental curves were fitted using a developed fitting program based on Fresnel equations [77,78,79,80,81,82,83] (Figure 4b–h). The obtained refractive index of the gold film was found to be in good agreement with previous studies [84,85], which yielded the *n*- and *k*-values of 0.276 and 3.897, respectively, with a thickness of 57.1 nm. The *n*- and *k*-values of the DA solution were the same as deionized water for concentrations less than 10 pM; after that, the *k*-value increased to 0.003. Meanwhile, the refractive index of CQD film was found to be 1.309 + 0i with a thickness of 13.72 nm. The real part of the refractive index of the CQD thin film decreased after contact with DA, as shown in Table 1. This decrease in the sensing layer’s refractive index after its exposure to DA solutions of varying concentrations was reflected in the blue shift of the SPR dips. It is worth noting that this interaction affected both the real part of the refractive index as well as the thickness of the sensor surface.

Indeed, a blue shift occurred in the SPR spectrum simultaneously with the morphological change in CQD thin film in the presence of DA. This blue shift elucidated morphological changes caused by DA adsorption, which resulted in CQD film tip truncation and a change in electron cloud density over the film surface [86]. This is completely consistent with the results of the AFM analysis. These findings confirm that the sensor film degraded where its thickness decreased from 13.72 nm to 9.30 nm and its roughness was decreased from 1.60 nm to 0.642 nm during DA adsorption on the surface of the film, although there is no strict linear correlation between the thickness and roughness of thin films. This is in agreement with other studies that reported that relation between the film thickness and its surface roughness [87,88].

**Figure 5:**
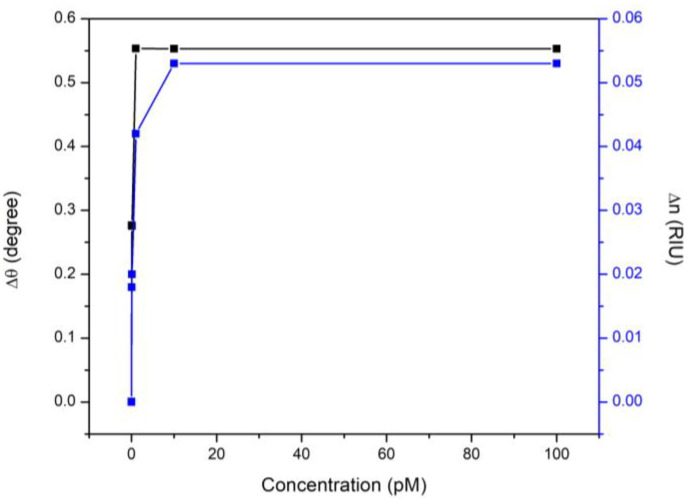
The increased change in the real part of the refractive index and resonance angle shift after the interaction of CQD film with different levels of DA.

It is clear from Figure 5 that increasing DA concentrations increased the change in *n*-value of the sensor film. This in turn increased the change in the resonance angle and indicated the high potential of the proposed sensor to detect extremely low levels of DA depending on the variation that occurred in the refractive index of the sensing layer.

### 3.3. Evaluation of Sensing Characteristics

**Figure 6:**
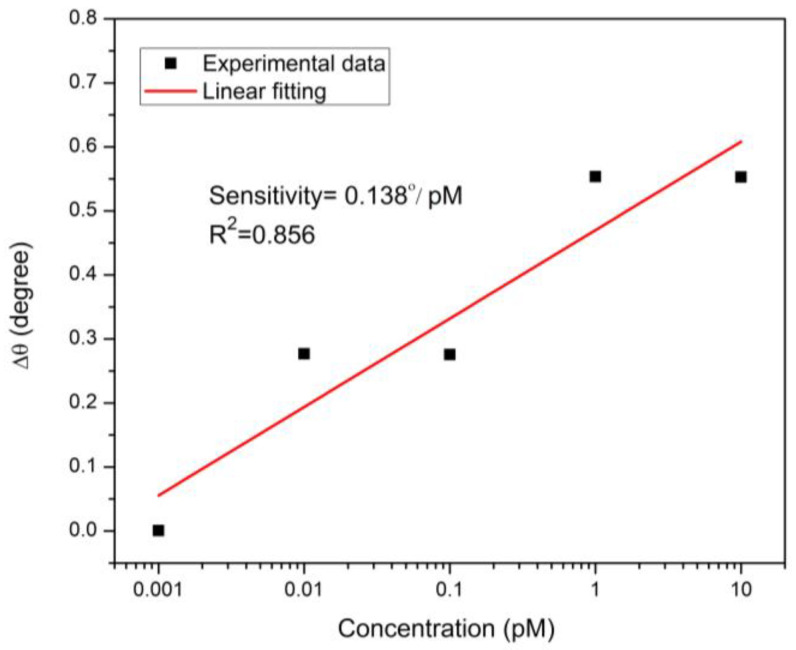
Linear fitting for CQD sensor film exposed to various concentrations of DA.

Considering that sensitivity is so important in evaluating the sensor performance, high sensitivity is always preferred. The sensitivity of SPR sensors depends on the configuration of the sensor, as well as surface roughness and homogeneity. The angular sensitivity of SPR sensors is defined as the change in resonance angle Δθ per the change in the target concentration [89,90,91,92,93,94]. Figure 6 depicts the relationship between DA solution concentration and the change in resonance angle for the CQD-based sensor. The good linear fitting demonstrates that this CQD film-based SPR sensor had a good sensitivity of 0.138°/pM for a DA level ranging from 0.001 to 100 pM with an R^2^ value of 0.856. 

This sensitive, simple, and label-free sensor has the ability to detect DA directly down to 0.01 pM. This lowest concentration of DA solution distinguished by the sensor from its baseline signal is defined as the limit of detection (LOD) [95,96], which is the lowest level of DA that was detected by carbon dot-based sensors, as shown in Table 2. This comparison highlights the sensor’s remarkable performance and emphasizes its efficiency in monitoring very low levels of DA without the need to use any additives or to functionalize CQDs.

Furthermore, the refractive index sensitivity $S_{RI}$ of this SPR sensor was also investigated. In angular interrogation mode, it is defined as the change in *Δθ* per the change in the real part of the refractive index of the sensor film and denoted as [114,115,116,117]:

(1) $$S_{RI}=\frac{\Delta \theta }{\Delta n}$$

**Figure 7:**
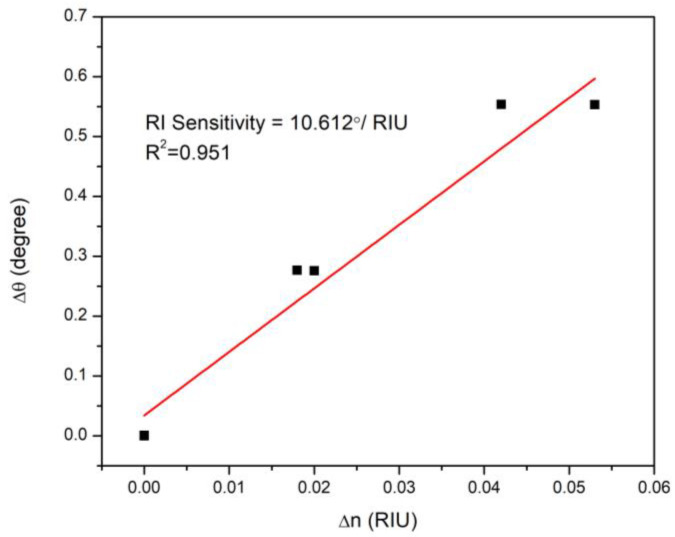
Linear fitting of the change in resonance angle versus the change in the sensor film refractive index exposed to gradually increasing doses of DA.

Figure 7 shows the linear fitting of the calculated values from experiments (*Δθ*) and theoretical fitting (*Δn*) listed in Table 1. The proposed sensor exhibited high refractive index sensitivity of 10.612°/ RIU (refractive index unit) that was obtained from the slop of the fitting line with R^2^ value of 0.951.

**Figure 8:**
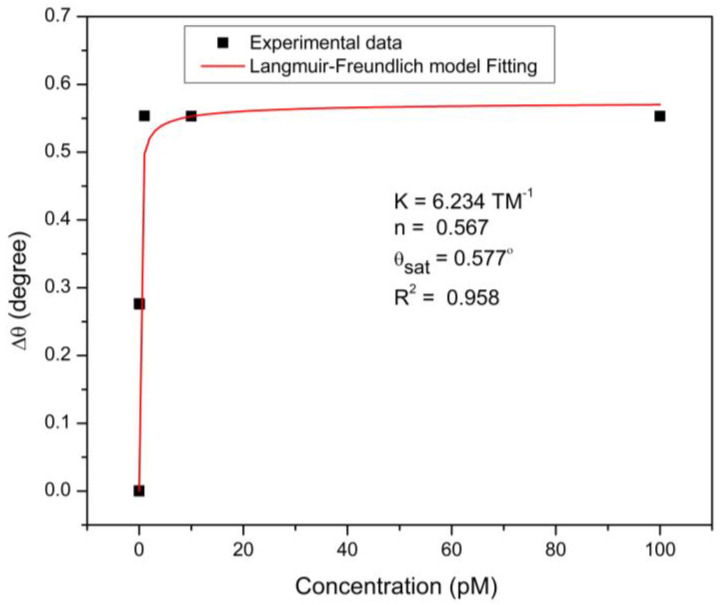
Experimental and fitted data to the Sips model for DA adsorption on CQD sensor film.

To investigate the binding affinity of the CQD-based sensor towards DA molecules, non-linear fitting based on the Langmuir and Freundlich isotherm model was applied to the experimental data, as shown in Figure 8. This model, known as the Sips model, combines both the Langmuir and Freundlich models, which give information on the heterogeneity in the adsorption behavior over a wide concentration range up to saturation, overcoming the Freundlich model’s limitation that appears at high concentrations of adsorbate. As a result, when the analyte concentration is low, the Sips model is reduced to the Freundlich model, whereas when the analyte concentration is high, it predicts monolayer adsorption and is indicative of the Langmuir model [118,119,120]. The Sips model is expressed as follows [121,122]:

(2) $$\Delta \theta =\frac{\Delta \theta_{max}\;K\;C^{n}}{1+KC^{n}}\;$$

where *K* is the Sips affinity constant, *C* is the concentration of DA solution, and *n* is the system heterogeneity index. The obtained correlation coefficient (R^2^ of 0.958) proves that Sips isotherm model was well fitted to the experimental results, with an affinity constant of 6.234 TM^−1^. The *Δθ_max_* value obtained from this model was very close to the experimental value (0.553°), and the Sips exponent value was 0.567.

The lowest full width half maximum (FWHM) value of 2.84704° was obtained for the detection of 1 fM DA, and the highest detection accuracy of 0.35124 (deg^−1^) was achieved for this level of DA, as shown in Table 3. Since the detection accuracy is inversely proportional to FWHM, the detection accuracy decreased by increasing DA concentrations. To explain this, increasing DA levels increased the truncation of the sensor film. Based on SPR dependance on the surface morphology, one of the key reasons for the blue shift in the SPR dips is that the induced dipoles were all out of phase. As the truncation in the sensor film increased and the number of faces of the nanoparticle increased, the main resonance underwent a blue shift owing to the augmentation in coulombic restoring force. Moreover, the truncation of the spherical nanoparticle into a multi-face structure caused the secondary SPRs to overlap, increasing the FWHM values, which in turn decreased the detection accuracy [123,124].

**Figure 9:**
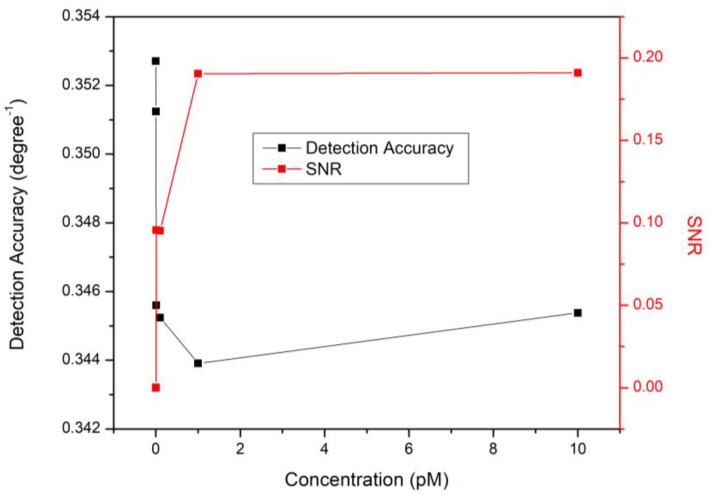
Detection accuracy and SNR of the DA SPR sensor based on CQD thin film.

It is critical to include the signal-to-noise ratio (SNR) while evaluating the sensor performance and quantifying its precision since it encompasses the impacts of resonance angle shift and detection accuracy. Figure 9 depicts the change in SNR and detection accuracy as a function of DA concentration. It is obvious that increasing DA levels resulted in noise reduction of the SPR signals and higher SNR values for the developed sensor. To explain this, increasing DA concentrations affected the refractive index of the sensor surface, causing the SPR dips to shift. SNR could be a binding affinity indicator since it is essentially dependent on the resonance angular shift [125,126].

## 4. Conclusions

To summarize, a CQD-based refractive index SPR sensor was developed and employed to detect DA. The performance of the proposed sensor was examined for different doses of DA solution. The experimental results showed that increasing DA levels resulted in blue-shifted SPR dips due to the adsorption of DA on the surface of the sensor film and the change in the morphology of the sensor film, which was confirmed by FTIR and AFM characterization and was proven subsequently by fitting the experimental SPR curves to theoretical ones based on Fresnel equations that made it possible to determine the optical parameters and thickness of the sensor film. This sensor exhibited an excellent sensitivity response of 0.138°/pM in a linear range from 0.001 to 100 pM and a high binding affinity of 6.234 TM^−1^. Because of its simple design, high sensitivity, and other detecting capabilities, this optical sensor has promising significance as a suitable platform for direct measurement of DA. The challenge of improving sensor chip stability throws out interesting possibilities for future work and the widespread use of nanomaterials.

## Figures and Tables

**Table 1:** The resonance angle, refractive index, and thickness values for CQD film determined by fitting experimental SPR curves to the theoretical, change in the real part of the refractive index *∆n*, and change in the resonance angle *∆θ*.

DA Concentration (pM)	**Resonance** **Angle** **(Degree)**	**Refractive Index of CQD Layer Exposed to DA**		**Thickness of CQD Layer** ***d*** **(nm)** **(±0.01)**	**Δ*n***	**Δ*θ***
		Real Part, *n* (±0.0001)	Imaginary Part, *k* (±0.0001)			
0	53.28702	1.309	0.000	13.72	0.000	0.00000
0.001	53.28647	1.309	0.000	13.72	0.000	0.00055
0.01	53.01033	1.291	0.000	12.82	0.018	0.27669
0.1	53.01144	1.289	0.000	12.70	0.020	0.27558
1	52.73346	1.267	0.000	10.20	0.042	0.55356
10	52.73401	1.256	0.000	9.30	0.053	0.55301
100	52.73401	1.256	0.000	9.30	0.053	0.55301

**Table 2:** Comparison of the proposed SPR sensor with other DA sensors using CQDs in terms of limit of detection and response range.

Material	Sensor	LOD	Linear Range	Reference
S, N-CQDs	Fluorescence	0.082 µM	0–50 µM	[97]
NB-CQDs	Photoluminescence	11 nM	0.1–70 µM	[98]
CQDs	Photoluminescence	0.2 mM	20–100 mM	[99]
N-CQDs	Fluorescence	50 nM	0.25–243 µM	[100]
CDs/TYR	Fluorescence	60 nM	0.206—131.8 µM	[101]
CDs@MIP	Fluorescence	1.7 nM	25–500 nM	[102]
CDs/CuNCs	Fluorescence	32 nM	0.1–100 µM	[103]
CDs-AuNCs	Fluorescence	2.9 nM	5–180 nM	[104]
S-CDs@Au NPs/Fe^+3^	Colorimetric chemical	0.23 μM	0.81–16.80 μM	[105]
DECDs-AuNPs	Fluorescence	0.037 μM	0.1–3 μM	[106]
		0.23 μM	0.5–3 μM	
Aptamer-CDs/NG	Fluorescence	0.055 nM	0.1–5 nM	[107]
N-CQDs/DA/Tyr/AA	Fluorescence	0.035 μM	0.01–15 μM	[108]
SiCDs	Fluorescence	56.2 nM	0.1–100 μM	[109]
CQDs/Au NPs	Fluorescent aptasensor	0.01 μM	0.05–250 μM	[110]
CDs@ZIF-8	Fluorescence	16.6 nM	0.1–200 μM	[111]
CDs-CS/GCE	Electrochemical	11.2 nM	0.1–30 μM	[112]
CDs	Fluorescence	33 μM	33–1250 μM	[113]
CDs	Electrochemical	4.6 nM	0.05–2 μM	[66]
H-CQDs	Fluorometric Colorimetric sensor	8 nM 163 μM	100–1000 μM 1000–100 mM	[69]
CQDs/Au	SPR	10 fM	1 fM-100 pM	This work

S, N-CQDs: sulfur and nitrogen co-doped carbon quantum dots; NB-CQDs: nitrogen and boron co-doped carbon quantum dots; N-CQDs: nitrogen-doped carbon quantum dots; CDs/TYR: carbon dots/tyrosinase hybrid; CDs@MIP: molecularly imprinted silica nanosphere-embedded carbon dots; CDs/CuNCs: carbon dots/copper nanoclusters dual-emitting nanohybrids; CDs-AuNCs: carbon dots/gold nanoclusters hybrid; S-CDs: S-doped carbon dots; DECDs-AuNPs: dual-emission carbon dots and gold nanoparticles; aptamer-CDs/NG: DA aptamer-labeled carbon dots and nano-graphite; SiCDs: aminosilane-functionalized carbon dots; ZIF-8: zeolitic imidazolate framework-8; H-CQDs: honey-based carbon quantum dots.

**Table 3:** The FWHM, detection accuracy, and SNR values of the CQD-based SPR sensor in response to varied DA concentrations.

DA Concentration (pM)	FWHM (Deg)	Detection Accuracy (Deg^−1^)	SNR
0.0000	2.83521	0.35270	0.00000
0.001	2.84704	0.35124	0.00019
0.01	2.89353	0.34559	0.09562
0.1	2.89652	0.34524	0.09514
1	2.90773	0.34391	0.19037
10	2.89538	0.34537	0.19099
100	2.95798	0.33806	0.18695

## Data Availability

Not applicable.
